# Accelerated Aging and the Life Course of Individuals Born Preterm

**DOI:** 10.3390/children10101683

**Published:** 2023-10-13

**Authors:** Audrey Bousquet, Keia Sanderson, T. Michael O’Shea, Rebecca C. Fry

**Affiliations:** 1 Department of Environmental Sciences and Engineering, Gillings School of Global Public Health, University of North Carolina, Chapel Hill, NC 27599, USA; aaudrey@live.unc.edu (A.B.); rfry@unc.edu (R.C.F.); 2 Department of Internal Medicine, School of Medicine, University of North Carolina, Chapel Hill, NC 27599, USA; keia_sanderson@med.unc.edu; 3 Department of Pediatrics, School of Medicine, University of North Carolina, Chapel Hill, NC 27599, USA

**Keywords:** prematurity, accelerated aging, allostatic load, lung disease, cardiovascular disease, dementia, cognitive decline, kidney disease, sarcopenia, immune senescence

## Abstract

Individuals born preterm have shorter lifespans and elevated rates of chronic illness that contribute to mortality risk when compared to individuals born at term. Emerging evidence suggests that individuals born preterm or of low birthweight also exhibit physiologic and cellular biomarkers of accelerated aging. It is unclear whether, and to what extent, accelerated aging contributes to a higher risk of chronic illness and mortality among individuals born preterm. Here, we review accelerated aging phenotypes in adults born preterm and biological pathways that appear to contribute to accelerated aging. We highlight biomarkers of accelerated aging and various resiliency factors, including both pharmacologic and non-pharmacologic factors, that might buffer the propensity for accelerated aging among individuals born preterm.

## 1. Introduction

Preterm birth, defined as birth before 37 weeks of gestation, affects about 15 million (11%) births worldwide each year and is the most frequent cause of infant mortality [1,2,3]. Individuals born preterm have a shorter lifespan than those born at term [4] and higher risks of chronic illness that contribute to mortality risk [5,6,7,8,9]. Such illnesses increase physical and emotional stress [10] and accelerate biological aging [11], resulting in an individual’s biological age exceeding their chronological age [12]. Biological age reflects the pace of aging as influenced by external and internal stressors such as environmental exposures or genetic factors [13].

Chronic illnesses to which individuals born preterm birth are at increased risk include cognitive [14,15], cardiovascular [5,16,17], pulmonary [6,18], metabolic [19,20], kidney [21], and psychiatric disorders [22,23], and these conditions often persist through the life course. Since chronic illnesses are associated with accelerating aging, it is possible that accelerated aging is a mechanism that links preterm birth and chronic health disorders across the life course [24]. Currently, a clear understanding of the impact of accelerated aging on individuals born preterm is lacking because few cohorts of preterm births have been followed beyond early adulthood.

**Figure 1:**
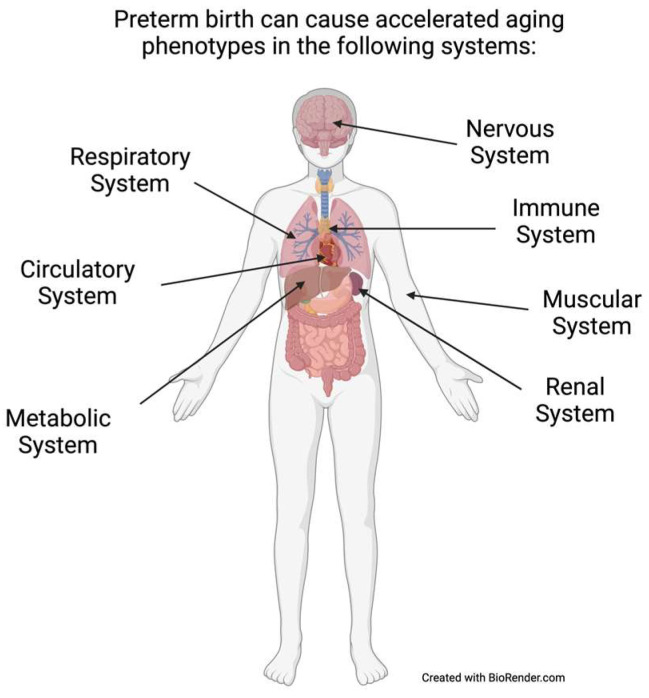
Body systems associated with accelerated aging.

In this paper, we review (1) phenotypes and biomarkers of accelerating age; (2) studies of associations between gestational age at birth or birth weight and phenotypes/biomarkers of aging; (3) biological mechanisms underlying aging; and (4) pharmacologic and non-pharmacologic risk and resiliency factors that could influence the pace of biological aging. We emphasize studies focused on individuals 18 years and older who were born very preterm (<32 weeks of gestation) or extremely preterm (<28 weeks of gestation) [25], while also including findings from cohorts of very low birth weight (VLBW; birth weight < 1500 g) and extremely low birth weight (ELBW; birth weight < 1000 g) neonates, which are comprised primarily of individuals born prematurely [26]. To illustrate the potential link between preterm birth and accelerated aging, we describe associations between preterm birth and accelerated aging phenotypes (AAP) within various organ systems, illustrated in Figure 1: (1) cardiovascular/circulatory; (2) metabolic–endocrine; (3) brain; (4) lung; (5) muscular system; (6) kidney; and immune systems.

Methods used in this narrative review were not prospectively specified. We limited our attention to papers published in English. We used PubMed and Google Scholar to identify papers with information that we regarded as relevant to the focus of our paper using the following search strategies: (1) [very low birth weight/extremely low birth weight/very preterm/extremely preterm] AND [cognitive impairment/cerebral palsy/brain MRI/brain volumes/lung/bronchopulmonary dysplasia/kidney/renal/immunity/hepatic/diabetes/obesity/cardiometabolic/blood pressure/hypertension/cardiovascular/metabolic/sarcopenia/aging], where each of the 4 terms listed in the first set of brackets was paired with each of the 11 terms in the second set of brackets; (2) biomarkers AND [aging/epigenetic clocks/telomere length/inflammation/oxidative injury], where biomarkers was paired with each of the terms listed in the brackets; and (3) senolytics. For the first set of searches, we reviewed the abstracts to identify the age of study participants and excluded studies that included participants younger than 18 years of age. In general, when searching for studies of “biomarkers and aging” and the search for senolytic drugs, we limited our attention to reviews that were available to us as full manuscripts from PubMed or Google Scholar. All associations and correlations that are described in this review were regarded by the original authors as statistically significant, except where we have specifically stated otherwise.

## 2. Results

### 2.1. Accelerated Aging Phenotypes and Biomarkers

#### 2.1.1. Prematurity or LBW and Cardiovascular Diseases

The risk of cardiovascular diseases increases with advancing age and also increases among adults born preterm [27,28,29]. This accelerated aging phenotype includes decreased arterial distensibility, hypertension, coronary heart disease, and heart failure. Frequently used in research studies of aging are non-invasively measured markers including systolic blood pressure (SBP) [30], diastolic blood pressure (DBP) [31], pulse pressure [32], and heart rate [33]. Elevated SBP (>140 mm Hg), DBP (>90 mm Hg), and heart rate (>90 beats per minute) tend to increase with chronological age and are associated with increased risk of cardiovascular death and/or all-cause mortality. Endothelial dysfunction, which elevates SBP, can be assessed non-invasively by measuring the hyperemic response to transient arterial occlusion; decreased hyperemia is indicative of endothelial dysfunction and is associated with advancing age [34,35,36]. Blood levels of homocysteine have also been used as a biomarker for cardiovascular risk; higher levels are associated with atherosclerosis and adverse cardiovascular events [37].

Many of these cardiovascular markers have been identified within populations of preterm or LBW individuals. In the HAPI (Health of Adults Born Preterm Investigation) cohort, adults (18–29 years old) who were born before 30 weeks of gestation had increased arterial stiffness and diastolic blood pressure, as compared to controls [38]. In a group of young adults born with VLBW, reactive hyperemia was decreased and SBP was increased, as compared to non-VLBW controls [39]. A systematic review of 10 studies of individuals born preterm or VLBW, concluded that preterm birth and VLBW were associated with elevated blood pressure [27]. In the ESTER (Preterm Birth and Early Life Programming of Adult Health and Disease) birth cohort from Finland, average blood pressure from 24 h monitoring was 5.5 mmHg higher in individuals born preterm than in term-born controls [40]. In the Cardiovascular Risk in Young Finns Study, average systolic blood pressure was 7.3 mmHg higher among adults born preterm (mean age of 41 years) [41]. Similarly, in an international collaboration involving 9 cohorts with 1571 VLBW adults and 777 term-born control adults, adults born VLBW, as compared to controls, have higher systolic [3.4 mm Hg; 95% confidence interval, 2.2–4.6] and diastolic blood pressure [2.1 mm Hg, 95% confidence interval 1.3–3.0] [42].

Hypertension and decreased arterial distensibility increase the risk of heart failure. A preterm cohort born in the United Kingdom (UK), with average gestational age at birth of 30 weeks and average age of 25 at follow-up, had larger left ventricular (LV) and LV wall thickness, with decreased LV internal cavity diameter and LV stroke volume, as compared to controls born at term [43]. In addition to hypertension, adults born preterm in the ESTER cohort had other cardiovascular risk factors including a higher percentage of body fat, higher waist circumference, and a higher rate of cardiometabolic syndrome [44]. Similarly, in the Aberdeen Children of the 1950s cohort, the lighter the participants were at birth, the greater the likelihood of them developing coronary heart disease or stroke [45]. Finally, adult males who were born at a low birth weight (LBW) in Helsinki, Finland had higher mortality due to coronary heart disease [46].

#### 2.1.2. Prematurity or LBW and Metabolic–Endocrine Diseases

Metabolic diseases including obesity and diabetes mellitus (DM) are major public health concerns, with obesity classified as a near-global epidemic [47]. Preclinical models and epidemiologic studies indicate associations between decreasing insulin sensitivity and shorter lifespan [48]. Higher insulin levels, reflecting lower sensitivity, are associated with greater age-related cognitive decline [49], and Alzheimer’s disease and type 2 diabetes mellitus share numerous mechanistic pathways [50]. A measure of insulin insensitivity over an extended timeframe is glycated hemoglobin (HgA1c). Obesity is associated with insulin insensitivity, so measures of obesity, such as body mass index and waist-to-hip ratio, are inexpensive methods that provide some insight into age-related changes in glucose and lipid metabolism. Dysregulated lipid metabolism is associated with age-related health disorders [51] and allostatic load [52]. Adults with type 1 diabetes mellitus have accelerated brain age as compared to controls without diabetes [53].

Measures of DM and obesity have been observed in preterm and LBW individuals. In a study of United Kingdom (UK) adults (aged 18–27), people born preterm (mean: 29 weeks gestation), as compared to those born at term, had higher levels of total and abdominal adipose tissue [54], risks factors for type 2 diabetes, hypertension, and dyslipidemia. In another sample, adults (34–38 years old) born preterm had lower insulin sensitivity than adults born at term [55]. In studies comparing ELBW adults with adults with normal birth weight, ELBW adults were more likely to develop dysglycemia (unstable blood sugar) [56] and had a higher percent of total body fat, fat in liver and pancreas, and subcutaneous fat [57]. In studies comparing VLBW adults with normal birth weight controls, VLBW adults had lower insulin sensitivity [58] and higher fasting concentrations of triglycerides in chylomicrons, very-low-density lipoproteins, and high-density lipoproteins, increasing the risk of cardiovascular disorders [20].

The Extremely Low Gestational Age Newborn (ELGAN) cohort is one of the largest and most diverse cohorts of individuals born extremely preterm. Although the ELGAN cohort has not yet been studied, this cohort exhibited an increasing frequency of obesity, from 2 years to 10 years to 15 years of age [59,60], and, in this cohort, obesity was associated with asthma [61].

#### 2.1.3. Prematurity or LBW and Brain Disorders

With advancing age, cognitive function declines [62] and the risk of dementia increases [63]. Cognitive functions that decrease with age include reasoning, spatial visualization, working memory, and processing speed. Total brain, grey matter, and white matter volumes also decrease with age [64]. Magnetic resonance imaging (MRI) can be used to estimate the brain age gap estimate (brainAGE) [65], defined as the difference between chronological age and age predicted from MRI data. Accelerated brain aging (estimated brain age > chronological age) has been associated with markers of aging, such as weaker handgrip strength, worse lung function, slower walking speed, lower fluid intelligence, and increased mortality risk [66].

Individuals born preterm have higher risks of cerebral palsy, a brain-related impairment that is about 80–90 times more prevalent among individuals born extremely or very preterm, as compared to those born at term [67,68], and 20 times more prevalent among those born VLBW as compared to those with normal birth weight [69]. The most prevalent brain-related disorder among individuals born preterm is cognitive impairment, identified with intelligence tests and assessments of executive function [70]. In a geographically based cohort of infants born in the United Kingdom before 26 weeks of gestation, 15% of young adults born extremely preterm had intellectual deficits, which was not found in any of the 64 controls [14]. Similar disparities in the prevalence of cognitive impairment between adults born preterm and those born at term have been reported in Norway [67] and Bavaria [15]. Although a precise understanding of the reason for this disparity in cognitive function between young adults born preterm and those born at term is lacking, adults born very preterm have decreased neural between-network connectivity at resting state [71], decreased brain volumes in deep grey matter structures [72], and reduced grey matter volumes in multiple brain regions [73], including the cerebellum [74,75]. In studies where both brain volumes and cognitive function have been assessed, reduced brain volumes have been correlated with lower cognitive function [73,74]. 

Adults born preterm are two times more likely to develop cerebrovascular disease [76], a risk factor for stroke. The risk of cerebrovascular disease is elevated among individuals with obesity, hypertension, or diabetes, which are common phenotypes among adults born preterm [77]. Preterm birth [78,79] and cerebrovascular disease [80,81] are associated with systemic inflammation. In the ELGAN cohort, neonatal systemic inflammation was associated with increased risks of cerebral palsy, learning and development deficiencies, and reduced white and grey matter volumes in the brain [82,83,84,85]. Fetuses with biomarkers of placental inflammation were more likely to exhibit neonatal systemic inflammation. With continued follow-up into middle adulthood, the ELGAN study provides the opportunity to evaluate the hypothesis that perinatal inflammation is associated with increased risks of cerebrovascular disease decades later [86].

#### 2.1.4. Prematurity or LBW and Lung Diseases

Lung function declines with age and, as a group, individuals born preterm have reduced lung function. In adults, lung function is most often evaluated by measuring airflow as the study participant exhales as rapidly and forcefully as possible. Among multiple measures of airflow, the most frequently used in epidemiologic studies is the forced expiratory volume in one second (FEV1), referring to the volume of gas exhaled during a forced expiration with maximal effort. 

The most prevalent pulmonary function abnormality associated with preterm birth is airway obstruction. Adults born preterm in a Norwegian cohort had lower FEV_1_ than controls born at term [87] and had greater airway constriction in response to methacholine exposure (hyperactive airways) than those born at term. Preterm birth is associated with oxidative and inflammatory lung injury [88], which in some cases resolves and in other cases evolves into a chronic lung disease referred to as bronchopulmonary dysplasia (BPD), the risk of which is inversely related to gestational age at birth [89]. Among adults born preterm, BPD is associated with decreased exercise tolerance [90], and, irrespective of whether they have BPD, adults born very preterm have impaired exercise capacity [91], worse lung function, and lower lung diffusion capacity for carbon monoxide [92]. Additional research is needed to understand whether adults born preterm are more likely to develop chronic obstructive airways, but such an association was found in a Swedish cohort of individuals born preterm or VLBW [93]. Studies focused on adults born with VLBW, rather than preterm birth, have found associations with lower FEV_1_ [94] and a higher likelihood of being hospitalized for a respiratory illness, i.e., respiratory infection, asthma, or respiratory failure [95].

#### 2.1.5. Prematurity or LBW and Sarcopenia

Sarcopenia refers to the age-related decline in both muscle mass and strength which is associated with an increased risk of falls and decreased ability to perform activities of daily living, independence, and quality of life [96,97]. Muscle mass can be measured in a number of ways, including computerized tomography (CT), magnetic resonance imaging (MRI), dual-energy X-ray absorptiometry (DXA), and bioimpedance analysis [98]. However, these methods are not well suited for large epidemiologic studies; in contrast, bioimpedance is inexpensive and portable. In large studies, the most widely used tool for measuring muscle strength (the magnitude of force generated) is a handgrip dynamometer to evaluate grip strength, which correlates with leg strength. The stair climb power test takes only a few minutes to complete and correlates well with other leg power impairment measures [99].

The risk of sarcopenia is increased by insulin resistance [100], which, as mentioned previously, is associated with preterm birth. Few preterm birth or LBW cohorts have evaluated biomarkers of sarcopenia beyond young adulthood. In a study of individuals 56–70 years old, born between 1934 and 1944 in Helsinki, Finland, birth weight was positively correlated with both lean muscle mass and grip strength, which are predictive of the degree of fragility [101]. Similarly, adults with ELBW born in Ontario, Canada had reduced grip strength at 23 years old when compared to adults born at a normal birth weight [102]. Thus, adults born preterm may be at increased risk of sarcopenia.

#### 2.1.6. Prematurity or LBW and Kidney Diseases

On average, humans are born with one million nephrons, or functional units, per kidney [103]. It is well established that the aging process significantly impacts the kidneys as a result of the oxidative stress from high vascular blood flow and metabolic activity. The hallmarks of kidney aging and chronic kidney disease include nephron loss, compensatory hypertrophy of remaining nephrons, kidney fibrosis, and total kidney volume loss [103,104,105]. Kidney biomarkers of aging and declining kidney function include serum creatinine or cystatin c (from which glomerular filtration rate can be estimated) and urinary protein. However, these biomarkers are less specific to aging and can be abnormal in the setting of an active or chronic inflammation in the kidney. An increasing number of studies are emerging that explore the use of Klotho and p21 expression in kidney biopsy tissue, plasma, and urine, and this may offer an opportunity to predict biological age in persons at high risk for kidney disease [106,107]. More research is needed to explore less invasive biomarkers which more specifically predict premature kidney aging.

Preterm birth or LBW are strong risk factors for the disruption of nephrogenesis and decreased nephron number (functional units of the kidney) [108]. As a result, children born preterm or with low birth weight, compared to infants born at term are about twice as likely to develop chronic kidney disease (CKD) over a lifetime [21]. In human autopsy kidney biopsy studies of infants born preterm, the same macro- and microscopic structural changes seen in a chronologically advanced age kidney can be seen in the kidneys of infants born preterm. For example, in a human kidney autopsy study, compared with gestational controls, preterm kidneys had greater percentages of morphologically abnormal glomeruli with significantly larger cross-sectional areas suggesting compensatory hypertrophy. Kidney volume loss is most commonly seen in adults who have chronologically advanced age, but abnormally reduced kidney volumes have also been shown to occur in individuals born extremely preterm [109].

In other human kidney disease states, aging biomarkers such as telomere length have been shown to be significantly shorter among individuals with diabetic kidney disease in comparison to age-matched controls without diabetic kidney disease [110]. Individuals born preterm have shorter telomere length in comparison to aged-matched persons born at term, and increased levels of this cellular aging biomarker are also associated with kidney fibrosis seen in diabetic kidney disease [111]. Klotho is an anti-aging protein expressed primarily in the kidney. It is downregulated in advanced chronologic age and has anti-inflammatory and anti-apoptotic properties which influence intracellular signaling pathways for aging biomarkers p53/p21^CIP1^ [112]. As a group, young adults born preterm have reduced urinary kidney α-klotho excretion indicative of reduced Klotho expression in the kidneys, as is also found in persons with chronic kidney disease [112,113].

In addition to findings demonstrating that reduced nephron number can be associated with senescence pathways and a premature aging phenotype (e.g., fibrosis) in the kidneys, individuals with kidney disease are also known to experience premature vascular calcifications and stiffness or vascular progeria that are also associated with biomarkers of aging. Adults with hypertension and kidney disease, as compared to those without these disorders, have higher expression of p16*^INK4a^* expression in kidney tissue [114]. Expression of p16*^INK4a^* in the kidneys of persons born preterm has not been studied, but since preterm birth is associated with hypertension, investigation of this protein in adults born preterm seems warranted [115].

#### 2.1.7. Immune Function Measures

Aging is associated with declining function of the immune system, increasing the risk of cancer and infectious diseases [116]. Levels of protein mediators of inflammation tend to increase with advancing age [117]. In epidemiologic studies of aging, the most often studied biomarkers are interleukin-6 and C-reactive protein. The levels of these proteins are inversely related to cognitive function in adults [118].

#### 2.1.8. Hepatic Measures

As compared to other organs, the liver shows less evidence of age-related decline in function, although the volume and blood flow are decreased in elderly adults [119]. Biomarkers of hepatic function include serum albumin and alkaline phosphatase [120].

### 2.2. Biomarkers of Mechanisms Contributing to Biological Aging 

**Figure 2:**
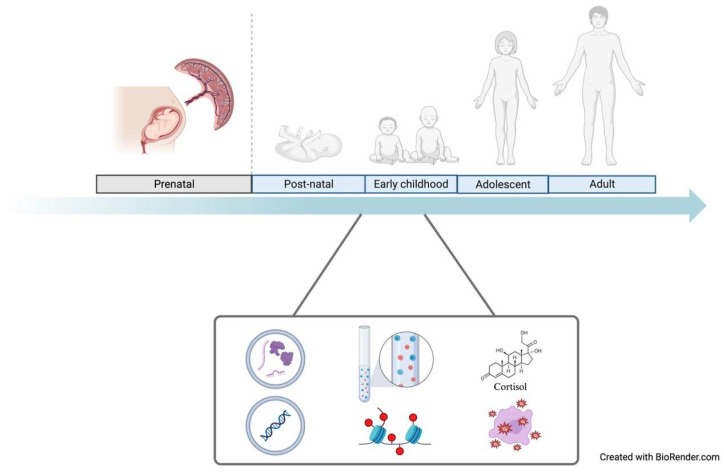
Biomarkers, such as reactive oxygen species, cortisol levels, and epigenetic profiles, can be assessed throughout the entirety of one’s life course using a variety of biospecimens.

Biomarkers of mechanisms contributing to biological aging (Figure 2) [121,122] could prove useful in designing interventions to prevent the acceleration of biological aging [123]. Below, we broadly classify biomarkers of aging mechanisms as related to either cellular aging or chronic stress.

#### 2.2.1. Cellular Aging Biomarkers

Biomarkers of cellular aging include epigenetic changes, genetic instability, telomere attrition, nuclear body disorders, mitochondria malfunction, proteostatic stress, metabolic alterations, cell cycle arrest, signaling pathway dysregulation, and senescence-associated secretory phenotype (SASP) [122]. Here, we emphasize epigenetic changes and telomere attrition.

Aging of individuals can be assessed using “epigenetic clocks” that are based on the level of DNA methylation in a set of genes for which methylation varies as a function of age” [124]. The first epigenetic clock, now known as Horvath’s epigenetic clock, was established by Bocklandt et al. in 2011 where they identified associations between chronologic age and the level of DNA methylation in specific sites in genes cells from saliva [125]. Subsequently, other epigenetic clocks have been developed for the placenta, blood, and saliva [123,126]. In a cohort of adults 30–35 years old (45 EBLW; 47 normal birth weight controls), males (n = 17) with EBLW had accelerated epigenetic age (*p* < 0.01), assessed with Horvath’s method, as compared to normal birth weight controls (n = 20) [13].

Telomeres are an evolutionary conserved complex consisting of a repeated nucleotide sequence and proteins situated at the ends of chromosomes to protect DNA from erosion [127]. Following mitotic division, telomeres shorten slightly, leading eventually to impaired cellular function. In preclinical models, telomere shortening has been implicated in kidney aging [128]. Telomere attrition is associated with decreased lifespan and increased risk of disease [129]. Adult males (18–27 years old) born very preterm had shorter telomere length than controls, suggesting accelerated biological aging [111].

#### 2.2.2. Biomarkers of Stress Responses

Response to stress has the potential to accelerate biological aging [11]. Biomarkers of stress responses include indicators of (1) allostatic load; (2) inflammation; (3) oxidative injury; and (4) activation of the hypothalamic–pituitary–adrenal axis. 

Allostatic load pertains to the cumulative stress experienced across an individual’s life course and the physiologic “aftermath” of adapting to that stress [130,131]. McEwen and Stellar refer to allostatic load as “the cost of chronic exposure to fluctuating or heightened neural or neuroendocrine response resulting from repeated or chronic environmental challenge that an individual reacts to as being particularly stressful” [132]. In a review of 58 studies of allostatic load, Juster et al. found the most frequently measured cardiometabolic biomarkers were systolic and diastolic blood pressures, waist-to-hip ratio, high-density lipoprotein cholesterol (HDL), and glycosylated hemoglobin (HbA1c); and the most frequently measures neuroendocrine biomarkers were 12 h urinary cortisol, epinephrine, and norepinephrine output, and serum dehydroepiandrosterone-sulphate (DHEA-S) [52]. Those 9 biomarkers, along with total cholesterol, were used in the longitudinal MacArthur Studies of Successful Aging cohort to derive a count-based allostatic load index [133]. In that cohort, higher allostatic load correlated with lower functioning, poorer cognitive performance, and weaker physical performance at baseline [133], and with higher all-cause mortality through 7 years of follow-up [134]. In addition to these anthropometric, neuroendocrine, metabolic, and cardiovascular markers, a small number of studies have included immune biomarkers, such as inflammation-related or proteins involved with coagulation. It has been suggested that infants born preterm inherit a heightened vulnerability to allostatic load and might be less capable of adapting to higher levels of stress [135].

Inflammation is a vital immune defense mechanism that can be activated by pathogens or damaged tissues [136]. As discussed above, aging is associated with the declining function of immune defenses against cancer and infectious diseases [116], but also with increasing levels of inflammation-related proteins [117]. Accelerated aging phenotypes, such as insulin resistance and increased adiposity [137] are pro-inflammatory. Preterm birth is associated with prenatal inflammation [138,139], and the risks of inflammatory neonatal diseases, such as necrotizing enterocolitis, sepsis, and BPD, are inversely related to gestational age [140]. 

Oxidative injury involves the damage of cells and tissues due to reactive oxygen species generated during a stress response, particularly inflammation [141]. Pregnancy complications, such as preeclampsia and intrauterine infection are associated with oxidative stress, and oxidative stress has been associated with neonatal complications of preterm birth [88,142,143]. Reactive oxygen species disrupt metabolism and have been implicated in the pathogenesis of numerous chronic diseases [144] and with shortening of telomeres [3].

The most widely used biomarker for hypothalamic–pituitary–adrenal (HPA) axis signaling is cortisol, which regulates physical and emotional stress responses [145]. Biomarkers of cortisol include the cortisol awakening response (cortisol released in the 30–45 min after waking) [146] and total salivary cortisol in 24 h, based on repeated measurements during a single 24 h period [147]. Total salivary cortisol evaluates diurnal variation [148]. Typically, cortisol levels are highest in the morning and decrease to their lowest levels at night [147]. During gestation, the HPA axis signaling [149] can be altered by maternal stress, increasing exposure of the fetus to glucocorticoids, with adverse consequences during the life course [150]. The HPA axis has not been studied in large cohorts of adults born preterm; however, among children born preterm, procedural pain during neonatal hospitalization was associated with altered HPA axis functioning at 7 years of age [151].

The Dunedin Study of individuals followed from early childhood into middle age illustrates how preterm cohorts could be studied to provide insights into potential links among preterm birth, accelerated aging, and shortened healthspan [120]. In that study, many of the biomarkers were selected based on their association with chronological age in the U.S. National Health and Nutritional Examination Study, and include blood pressure, hemoglobin A1c, total cholesterol, C-reactive protein, Cytomegalovirus IgG, creatinine, blood urea nitrogen, albumin, alkaline phosphatase, and forced expiratory volume in one second (pulmonary). Other biomarkers/phenotypes that were assessed in the Dunedin cohort were VO2Max (a measure of cardiorespiratory fitness), waist-to-hip ratio, body mass index, leukocyte telomere length, periodontal disease, white blood cell count, and lipid profile [120].

### 2.3. Resiliency Factors and Moderators for Accelerated Aging

Preclinical and human epidemiologic [152] studies provide support for the concept that the pace of aging is modifiable. Aging involves overlapping processes with marked connectedness, and when developing interventions to slow aging and extend healthspan, it is critical to address these highly intertwined processes, including metabolism, epigenetics, inflammation, adaptation to stress, proteostasis, stem cells and regeneration, and macromolecular damage [153]. Although medical research has typically targeted diseases separately, the functioning of individual systems is influenced by the health of other systems. Therefore, interventions for aging must consider the effects on multiple organ systems and morbidities. Interventions to modify aging include pharmacologic approaches, environmental changes, and enhancement of specific lifestyle factors, such as physical activity, diet, social relationships, and sleep [154].

Accumulating evidence suggests that non-pharmacological approaches that promote social health and cognitive activity can decrease the pace of cognitive decline [155,156,157]. “Social health” refers to human capacities to engage in social activities and structural and functional social networks. Cross-sectional studies have identified associations between higher social health factors and cognitive activities, larger total brain volumes and hippocampal volumes, and the lower frequency of white matter hyperintensities [158], providing some support for the biological plausibility linking higher social health factors and/or cognitive activities and decreased pace of brain aging [159].

Improvements in physical exercise and sleep quality are additional avenues for interventions to moderate the pace of aging. More frequent physical exercise and adequate sleep are associated with better health [160]. Exercise is an adjunctive therapy for various health outcomes, including obesity, diabetes, hypertension, and coronary heart disease [161]. Sleep promotes restorative functions which become increasingly important with age [162].

Alterations in dietary intake of micronutrients and calories may lower the risk of chronic diseases associated with accelerated aging [163,164]. Deficiencies of vitamins B_12_, B_6_, C, and E, folic acid, niacin, iron, and zinc can increase the risk of cancer [164], and risk can be lowered by increased consumption of fruits and vegetables. In preclinical models, reduced caloric intake can lengthen lifespan [165]. Caloric restriction becomes increasingly important as individuals age, metabolic rate decreases, and body fat increases [166]. Metabolic rate can be increased by maintaining healthy muscle mass levels and physical exercise [167].

Multiple aspects of the environment are potential targets for interventions to extend healthspan; perhaps the most obvious contributors are air pollution and climate change. Air pollution is largely caused by anthropogenic activities and exposure is associated with adverse health outcomes including difficulty breathing, birth defects, and cancer [168]. Gestational exposure to particulate matter (PM) with a diameter of ≤2.5 μm is associated with telomere shortening in cord blood and placenta, suggesting that molecular longevity could be negatively impacted by poor air quality [169]. Climate change is indirectly associated with aging through its effects on air pollution, heat stress, malnutrition, and vector-born illnesses [170].

Pharmacological approaches currently under investigation include interventions to reduce chronic inflammation and metabolic dysregulation [153], strategies to enhance vaccine efficacy in the elderly [159], therapies targeting senescent cells [171], and therapies to reduce the senescence-associated secretory phenotype [172]. In mice, rapamycin, metformin, and acarbose extend lifespan [173].

## 3. Conclusions

Although adverse health outcomes among adults born preterm are well-documented, knowledge gaps remain. Very few studies of extremely preterm individuals have collected detailed information on aging biomarkers and phenotypes, and few have evaluated biological mechanisms that could accelerate the pace of aging. For further studies of these research areas, study designs could include recruitment of new cohorts and continued evaluation of existing cohorts using serial measures of the exposome (aggregated index of environmental exposures; e.g., toxic metal exposures and air pollution), cellular and stress biomarkers, and accelerated aging phenotypes. Analyses could evaluate links among prenatal, perinatal, and postnatal exposures and biomarkers and mechanisms of aging. If, as we posit, adults born preterm are predisposed to accelerated biological aging, knowledge of biomarkers and mechanisms of aging could inform the design of interventions to moderate the pace of aging and thereby increase healthspan among the millions of individuals who are born prematurely each year.

## Data Availability

Not applicable.
